# Plasma proteome changes associated with refractory anemia and refractory anemia with ringed sideroblasts in patients with myelodysplastic syndrome

**DOI:** 10.1186/1477-5956-11-14

**Published:** 2013-04-08

**Authors:** Pavel Májek, Zuzana Riedelová-Reicheltová, Jiří Suttnar, Klára Pečánková, Jaroslav Čermák, Jan E Dyr

**Affiliations:** 1 Institute of Hematology and Blood Transfusion, U Nemocnice 1, Prague 2, 128 20, Czech Republic

**Keywords:** Myelodysplastic syndrome, Refractory anemia, Refractory anemia with ringed sideroblasts, Proteome changes, Alpha-2-HS-glycoprotein

## Abstract

**Background:**

Refractory anemia and refractory anemia with ringed sideroblasts are two myelodysplastic syndrome (MDS) subgroups linked with anemia. MDS is a group of heterogeneous oncohematological bone marrow disorders characterized by ineffective hematopoiesis, blood cytopenias, and progression of the disease toward acute myeloid leukemia. The aim of this study was to search for plasma proteome changes in MDS patients with refractory anemia and refractory anemia with ringed sideroblasts.

**Results:**

A total of 26 patient and healthy donor plasma samples were depleted of fourteen high-abundant plasma proteins, separated with 2D electrophoresis, and statistically processed with Progenesis SameSpots software. 55 significantly differing spots were observed and corresponded to 39 different proteins identified by nanoLC-MS/MS. Changes in the fragments of the inter-alpha-trypsin inhibitor heavy chain H4 protein were observed. Using mass spectrometry-based relative label-free quantification of tryptic peptides, there were differences in alpha-2-HS-glycoprotein peptides, while no differences were observed between the control and patient sample groups for retinol-binding protein 4 peptides.

**Conclusions:**

This study describes plasma proteome changes associated with MDS patients with refractory anemia and refractory anemia with ringed sideroblasts. Changes observed in the inter-alpha-trypsin inhibitor heavy chain H4 fragments were in agreement with our previous studies of other MDS subgroups: refractory cytopenia with multilineage dysplasia and refractory anemia with excess blasts subtype 1. Mass spectrometry-based relative quantification of retinol-binding protein 4 peptides has shown that there are differences in the modification of this protein between refractory anemia with excess blasts subtype 1 patients and MDS patients with refractory anemia and refractory anemia with ringed sideroblasts. Alpha-2-HS-glycoprotein seems to be a new potential MDS biomarker candidate.

## Introduction

Anemia, one of the most common blood disorders, is usually caused by the decrease of red blood cells. Thus, the oxygen-carrying capacity of the blood is decreased, and anemic patients consequently feel tired and suffer from other consequences of hypoxia. As one of the most common causes of anemia is bleeding, an iron deficiency, underproduction, or excessive destruction of red blood cells also occur.

Refractory anemia (RA) and refractory anemia with ringed sideroblasts (RARS) are two myelodysplastic syndrome (MDS) subgroups linked with anemia, according to the World Health Organization (WHO) classification of MDS [1]. Both RA and RARS are characterized by dysplasia limited only to the erythroid lineage; by less than 5% of blasts located in the bone marrow, and with limited response of the anemia to treatment.

Very little is known about plasma protein alterations occurring in myelodysplastic syndrome. Even though there are several proteomic studies dealing with MDS [2-4], including our previous studies of refractory cytopenia with multilineage dysplasia (RCMD) [5] and refractory anemia with excess blasts subtype 1 (RAEB-1) [6], there is still a lack of data from different MDS subgroups that would enable comparison of these subgroups. The possibility to find proteins that change either in general in MDS, or just within specific subgroups, could benefit our knowledge of the molecular mechanisms and (patho)physiology of MDS; and these results might thus influence the diagnosis, treatment, and prognosis of the disease.

Although there are many proteomic methods and techniques (e.g. chromatography, mass spectrometry, protein biosensors [7-9]) that offer the possibility to look for protein alterations in biologic samples, 2-D electrophoresis is usually the first choice despite some disadvantages. Nevertheless, there is no 'all purpose' proteomic method, and each one has its own limitations. The preference for 2-D electrophoresis in the first level of proteome exploration results from the possibility to observe within the analysis, not only proteins which change in the concentration, but also those posttranslationally modified. It has been shown that not only plasma protein levels change, but also the presence of specific protein isoforms, which may be of interest [10].

The aim of this proteomic study has been to explore the plasma proteome of MDS patients with refractory anemia and refractory anemia with ringed sideroblasts, and to search for possible protein alterations in comparison with healthy controls.

## Methods

A total of 10 patient plasma samples (7 RA and 3 RARS patient samples were included in the patient group further denoted as RA-RARS) and 16 healthy controls have been investigated in this proteomic study. The diagnosis of RA and RARS was established according to the WHO classification criteria [1]. The age of the patients ranged from 50 to 89 years, and there were 4 males (40%). Sex-matched, healthy control donor age ranged from 21 to 35, and there were 6 males (38%). All individuals tested agreed to participate in the study on the basis of an informed consent. All samples were obtained and analyzed in accordance with the Ethical Committee regulations of the Institute of Hematology and Blood Transfusion.

150 μl of diluted plasma (1:3 in MARS depletion buffer) was used to deplete high-abundant plasma proteins (MARS Hu-14 column; Agilent, Santa Clara, CA, USA). This corresponded to the volume of 37.5 μl of undiluted plasma used for each sample in this study. Blood sample collection, plasma depletion, and sample processing and storage were described in detail in our previously published literature [5,6]. 2D electrophoresis, image analysis, protein in-gel digestion, and mass spectrometry identification of proteins were also performed as described in previous literature [10-12]. Briefly, isoelectric focusing (IPG strips pI 4–7, 7.7 cm) was followed by SDS-PAGE (8 × 10 cm, 10% resolving gel, 3.75% stacking gel, 30 mA/gel), gels were scanned and processed with Progenesis SameSpots software (Nonlinear Dynamics, Newcastle upon Tyne, UK) that computed fold and p-values of all spots using one way ANOVA analysis, and Principal Component Analysis (PCA) was performed. PCA was performed using only the spots of statistical significance (based on 2D SDS-PAGE) employed for protein identification. PCA was used to assess whether grouping of patients and healthy controls based on the 2D SDS-PAGE results reflects their stratification using clinical diagnosis. No technical replicates were used for 2D SDS-PAGE (non-pooled individual samples of patients and donors were used only). MS/MS mass spectrometry (HCT ultra ion-trap mass spectrometer with nanoelectrospray ionization; Bruker Daltonics, Bremen, Germany) coupled to a nanoLC system (UltiMate 3000; Dionex, CA, USA) was used to perform MS analysis. Mascot (Matrix Science, London, UK) was used for database searching (Swiss-Prot). To eliminate peptide carry-over between HPLC separations, cleaning runs were performed before and after each sample run. Two unique peptides (with a higher Mascot score than the minimum for identification, p < 0.05) were necessary to successfully identify a protein.

Relative label-free protein quantification was used to compare tryptic peptide levels of alpha-2-HS-glycoprotein (A2HSG) and retinol-binding protein 4 (RBP4) in the patient and control groups. Plasma proteins were digested using modified protocols of acetonitrile precipitation of plasma proteins and trypsin digestion, as described by Kay *et al.*[13]. Briefly, to the 50 μl of sample plasma, 100 μl of water, and 225 μl of acetonitrile were added, and then sonicated twice for 10 min with vortexing between sonications. After centrifugation (10 min, 12000 × g) 300 μl of supernatant were transferred into a new Eppendorf tube and completely dried. Pellets were then resuspended in 40 μl of 0.1 M NH_4_HCO_3_, 5 μl of 0.1 M DTT were added, and the samples were incubated for 1 hr at 60°C. After the samples cooled, 5 μl of 0.1 M iodoacetamide were added, and the samples were incubated at room temperature in the dark for 30 min. Trypsin digestion was started by adding 4 μl of trypsin (200 μg/mL) in 50 mM acetic acid and digested at 37°C overnight. The reaction was stopped with the addition of 6.5 μl of 1% formic acid. Samples were centrifuged (10 min, 12000 × g), filtered, and 20 μl of each sample was loaded for LC-MS/MS analysis. Two A2HSG peptides were monitored: HTFMGVVSLGSPSGEVSHPR with precursor ion 694.7 m/z (charge 3+) and product ion 1109.5 m/z (y11); and EHAVEGDCDFQLLK with precursor ion 830.9 m/z (2+) and product ion 1095.5 m/z (y9). Peptides, precursor, and product ions were chosen on the basis of MS/MS analysis (previous protein identification). RBP4 peptides were the same as monitored in our previous publication describing plasma proteome changes in RAEB-1 [6]: FSGTWYAMAK with precursor ion 589.3 m/z (2+) and product ion 785.3 m/z (y6); and YWGVASFLQK with precursor ion 599.9 m/z (2+) and product ion 849.5 m/z (y8). The nanoLC-MS/MS system was the same as described above, but using a 60 min 0-70% acetonitrile non-linear gradient, with a precursor selection window width of 2 Da. Extracted ion chromatograms of the product ions were generated with an ion m/z width of ± 0.5 Da, and peak areas were calculated after automatic integration using DataAnalysis software (version 4.0; Bruker). Each sample was measured twice, and a *t*-test was used for statistical analysis. All sample results were validated according to MS/MS spectra and retention times [14]. Blank runs were performed before and after each sample run to eliminate peptide carry-over.

Western blotting was performed as described previously [10]. Briefly, proteins were transferred from a gel to a PVDF membrane (10 V constant voltage for 1 hr), the membrane was then incubated with a blocking buffer (3% BSA in PBS) at 4°C overnight, rinsed, and incubated with an anti-ITIH4 primary antibody (1:2,000 dilution) (Abnova, Taipei, Taiwan) or an anti-RBP4 primary antibody (1:2,000 dilution) (Abcam, Cambridge, UK), at 30°C for 45 min. Then the membrane was incubated with the secondary antibody, rabbit anti-mouse IgG antibody conjugated with peroxidase (for ITIH4 detection, 1:60,000 dilution) (Sigma-Aldrich, Prague, Czech Republic) or goat polyclonal anti-rabbit IgG antibody conjugated with peroxidase (for RBP4 detection, 1:10,000 dilution) (Abcam, Cambridge, UK), at 30°C for 45 min. After rinsing, a chemiluminescent substrate (SuperSignal West Pico; Thermo Scientific, Waltham, MA, USA) was added to the membrane for 5 min; and an appropriate film exposure (Amersham Hyperfilm ECL; GE Life Sciences, Piscataway, NJ, USA) was performed. In total, 6 patient (3 RA and 3 RARS) and 6 sex-matched control samples were used for western blotting; pooled patient and control samples were used in addition.

The alpha-2-HS glycoprotein plasma level was measured using a commercial ELISA kit (Abcam, Cambridge, UK). Sex-matched samples were measured according to manufacturer instructions; results were expressed as means ± standard deviations. An unpaired *t*-test was used for the comparison of plasma levels between RA-RARS and patient groups.

## Results

**Figure 1:**
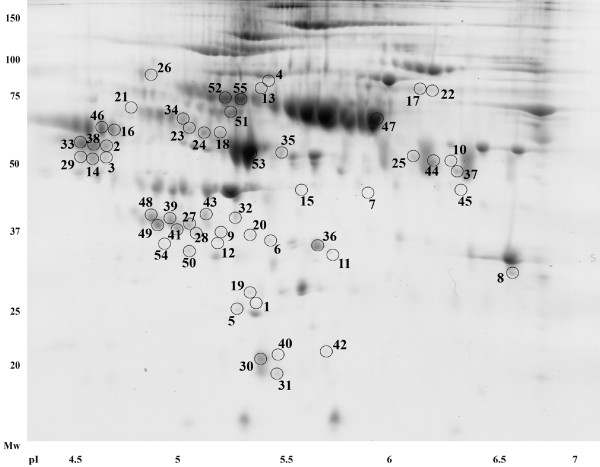
**Positions of significantly differing spots on a 2D gel.** Positions of all 55 spots that were found to differ significantly (p < 0.05) when RA-RARS patients were compared to healthy controls. A 2D gel of a patient sample was used as the illustrative gel to display spot positions.

**Figure 2:**
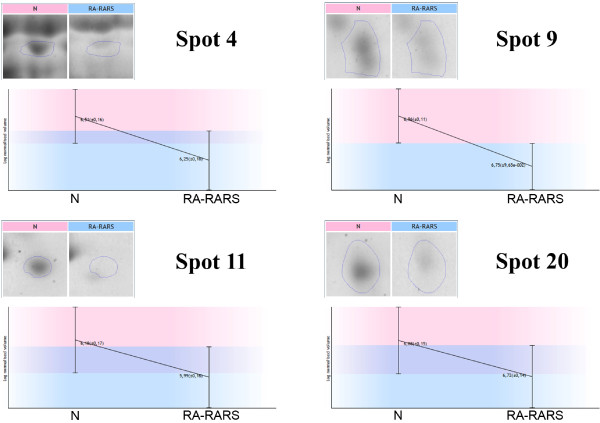
**An example of four differing spots.** This figure represents an example of four differing spots (4, 9, 11 and 20). Expression profiles with the logarithms of spot normalized volumes and their standard deviations for RA-RARS patient and healthy control groups are shown.

2-DE gels of all samples were prepared for the experiment, and scanned 2-DE gel images were divided into patient (n = 10) and control (n = 16) groups. Comparing these two groups we found 55 unique spots that differed significantly (p < 0.05) in normalized volumes (Figure 1). An example of four differing spots is shown in Figure 2. Proteins in 49 spots were identified, which corresponded to 39 different proteins. The list of all spots, including ANOVA p-values, their multiplication (fold value), protein identification with the number of identified peptides (unique peptides above the identity threshold score), protein accession number (Swiss-Prot), and the sequence coverage, is summarized in Table S1 (See Additional file 1: Table S1).

**Figure 3:**
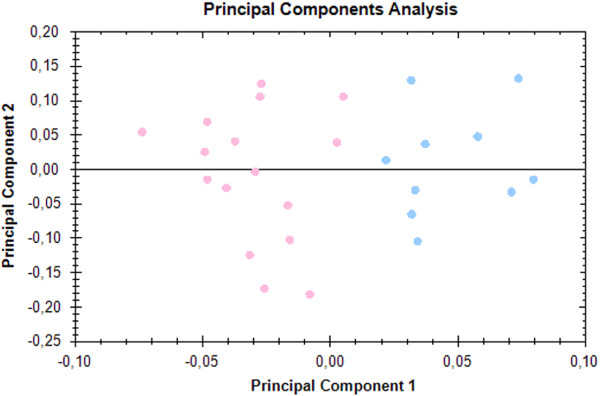
**Principal Component Analysis.** Analysis was based on spots that significantly differed according to the mentioned statistical criteria (p < 0.05, ANOVA), and was performed to assess whether grouping of patient and healthy controls based on 2D SDS-PAGE reflects their stratification using classical clinical diagnosis. PCA showed the separation of all samples into two aggregates that corresponded to the RA-RARS (blue dots) and healthy control groups (pink dots).

Principal Component Analysis (PCA) was performed making use of Progenesis SameSpots software employing only the spots of statistical significance (based on 2D electrophoresis). PCA showed an obvious separation of all samples (2-DE gels) into two aggregates corresponding to the patient (blue dots) and the control (pink dots) groups, respectively (Figure 3).

**Figure 4:**
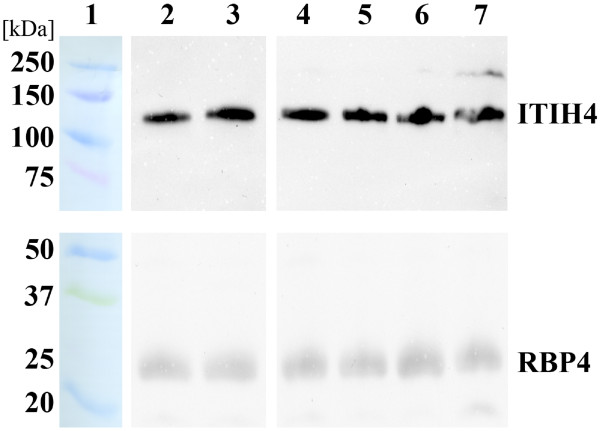
**RBP4 and ITIH4 western blot analysis.** Illustrations of 1D SDS-PAGE/Western blot analysis of ITIH4 (the top section; 10% resolving gel) and RBP4 (the bottom section; 15% resolving gel) are shown. Lane 1: molecular weight marker (kDa); lanes 2 and 3: RA-RARS patient (2) and control (3) pooled samples; lanes 4 to 7: an example of individual male (4, 5) and female (6, 7) samples of RA-RARS patients (4, 6) and healthy controls (5, 7).

In several spots (4, 9, 11, 12, 20, 27, 28, 41, 52, and 55), fragments of inter-alpha-trypsin inhibitor heavy chain H4 (ITIH4) was observed. The molecular weights of the spots, as estimated using 2-DE, corresponded to both 35 kDa ITIH4 (spots 9, 11, 12, 20, 27, 28, and 41) and 70 kDa ITIH4 (spots 4, 52, and 55) fragments. Moreover, when comparing the sequences of ITIH4 identified peptides (using MS/MS) in all spots with the observed fragmentation, these peptides corresponded to both 35 and 70 kDa ITIH4 processed proteins. Significantly differing spots containing uncleaved ITIH4 were not observed. ITIH4 expression was assessed by western blot analysis; a single band of more than 100 kDa of molecular weight was observed (Figure 4). This band corresponded to the uncleaved ITIH4, and no difference in ITIH4 expression between the patient group (n = 6) and the control group (n = 6) was observed. Using a monoclonal anti-ITIH4 antibody capable of detecting uncleaved ITIH4 and 70 kDa ITIH4 fragment, we did not observe the 70 kDa ITIH4 fragment (the antibody did not act against the 35 kDa ITIH4 fragments).

RBP4 expression was assessed by western blot analysis as described above; the same patient (n = 6) and sex-matched control (n = 6) samples as for ITIH4 western blot analysis were used. No difference was observed between patient RA-RARS and healthy control groups when comparing individual or pooled samples (Figure 4).

The data obtained using 2-DE cannot provide information on the cause of the observed change – plasma level change or posttranslational modification (PTM). Comparisons of different peptides of the same protein in two different groups (patient and control) using relative quantification may provide additional information – at least two unique fold change values in two different peptides can prove the presence of PTM(s), which would have influenced the results observed using 2-DE (based on spot volume changes). As co-identification of other proteins within a spot impairs data interpretation, proteins with unique identifications in their spots, together with satisfactory p-values, were considered for relative quantification. Moreover, we focused on proteins possibly related to MDS and its pathophysiology. Two proteins satisfied these criteria, and relative label-free protein quantification was used to compare tryptic peptide levels of alpha-2-HS-glycoprotein (A2HSG) and retinol-binding protein 4 (RBP4) in the patient (n = 10) and control (n = 10) groups. The samples were the same as used for 2-DE, and the control samples were sex-matched with the patient group samples. Quantification of A2HSG showed different fold change values for its peptides; the average peak area value was increased by more than two-fold in the MDS group (2.54-fold, no statistical significance) when compared with the control group for 694.7/1109.5, while there was no obvious difference for 830.9/1095.5 (0.95-fold decrease) in the MDS group when compared to the control group. For RBP4 peptides, there were observed no differences between the control and the patient sample groups for both peptides: 0.92-fold and 1.12-fold difference for 589.3/785.3 and 599.9/849.5, respectively. The results are summarized in Table 1.

**Table 1:** Relative label-free quantification of proteins

**Protein**	**Peptide sequence**	**Precursor ion [m/z]**	**Precursor charge**	**Product ion [m/z]**	**Product ion**	**fold (MDS/N)**
A2HSG	HTFMGVVSLGSPSGEVSHPR	694.7	3+	1109.5	y11	2.54
A2HSG	EHAVEGDCDFQLLK	830.9	2+	1095.5	y9	0.95
RBP4	FSGTWYAMAK	589.3	2+	785.3	y6	0.92
RBP4	YWGVASFLQK	599.9	2+	849.5	y8	1.12

A2HSG (alpha-2-HS-glycoprotein), RBP4 (retinol-binding protein 4), no fold value was found to be significantly different.

The plasma level of A2HSG was measured for all patient RA-RARS samples (n = 10) and sex-matched control samples (n = 10): 471 ± 38 μg/ml (RA-RARS) and 562 ± 28 μg/ml (control). This corresponds to 1.2-fold decrease of A2HSG plasma level in the patient group. The difference was found to be statistically significant (*t*-test, p = 1.3 × 10^-5^).

## Discussion

This paper represents the first report on plasma proteome changes using 2-DE in MDS patients with RA and RARS subgroups of MDS. Both RA and RARS are closely related subgroups of MDS, and according to Malcovati *et al.*[15] are characteristic with a very low risk of transformation into acute myeloid leukemia and good predicted survival. According to WHO Classification-Based Prognostic Scoring System (WPSS) for MDS [15], there are different risk categories – both RA and RARS belong to the very low risk group. Our previously studied groups RCMD [5] and RAEB-1 [6] belong to low and intermediate risk groups, respectively. Therefore, including this study, there are three different MDS risk groups that can be compared so far, and it is a future task to explore proteome changes in the high risk RAEB-2 subgroup. When the results of all of the three so-far-studied MDS subgroups are compared in general, it is interesting that there is no substantial difference in the numbers of observed changes; there were 61, 56, and 55 significant changes observed in RCMD, RAEB-1 and RA-RARS, respectively. Despite differences in the number of samples in the studied subgroups, we expected an increase in the number of observed changes in plasma proteome correlating to an increasing WPSS score of the studied subgroups. It seems that it is rather an increase in ‘a depth of changes’ than in the number of changes. Thus, it is important to look for changes in the initial step (selection of candidate proteins); however, it seems probably even more important to profile protein changes (whether caused by protein level changes or PTMs) in different MDS subgroups in the future. Another issue that should be noted is the choice of the control group, which differs in age range when compared to the MDS patient group. Optimally, the control group should match for as many parameters as possible. As MDS usually occurs in elderly patients, the control group should be of similar age range. However, this matching may contribute to other limitations – the primary difficulty is to find healthy individuals of older age who do not suffer from other diseases (diabetes, hypertension etc.) which may significantly affect the obtained results. Further, MDS is not exclusively limited to elderly patients only. Considering that, we decided to compare our patient group with a healthy control group of lower age range to possibly observe all the changes that would occur. Moreover, we used the same characteristic for the control group as in our previous proteomic studies of MDS patients [5,6] to be consistent in the results and their interpretation.

When compared to our previous proteomic studies characterizing changes in the plasma proteome of patients with RCMD [5] and RAEB-1 [6], there is agreement in some results. One of the most evident is the presence of fragmentation of the ITIH4 protein. Normalized volumes of spots containing ITIH4 fragments corresponding to 35 kDa ITIH4 were decreased in RA-RARS, as well as in both the RCMD and RAEB-1 groups, when compared to the control groups. A 70 kDa ITIH4 fragment was identified in the RA-RARS and RCMD groups with the same characteristics of the changes of spot volume (decrease); however, it was only co-identified in spots together with other proteins and thus its interpretation would be speculative. It remains a question whether ITIH4 fragmentation (or modification) could be MDS specific – comparisons of MDS subgroups together with different control groups (non-MDS cytopenias etc.) would be welcome. Even though ITIH4 fragmentation has been found to be potentially associated with MDS [3], it has been also found in other malignant diseases [16,17]. Therefore, ITIH4 fragments could in general be considered as a cancer marker, but not as a marker specific to MDS. Nevertheless, there should be other more specific proteomic data to definitively draw such conclusions; the fragmentation pattern should be elucidated, as well as the possible influence of PTMs. For example, the 70 kDa ITIH4 fragment was not observed in RA-RARS, RAEB-1, or RCMD (using western blot analysis) even though the antibody used should have detected it. This could suggest that the fragmentation did not correspond to that induced by kallikrein (which processes the plasma ITIH4 and cleaves it to produce 35 and 70 kDa fragments). This notion may be supported by the fact that fragments found in proteomic study by Chen *et al.*[3] using mass spectrometry plasma profiling were smaller than 35 or 70 kDa. Inflammation could be another factor that influences ITIH4 alterations, as this protein is known to belong to the group of acute phase proteins.

A2HSG has been found to be related to several functions – endocytosis, opsonization, or bone tissue formation [18-20]. This protein has been localized in the bone marrow matrix, and is believed to influence bone marrow development and function [21]. It has been also shown that A2HSG is linked to inflammation, and is one of the acute phase proteins, which may be regulated either positively (in some cases) or negatively during an acute phase reaction [22-24]. A2HSG is also supposed to be involved in diabetes [25] and insulin resistance [26], the regulation of protease activity [27], the development of atherosclerosis [28], etc. It has been shown that A2HSG regulates tumor growth in mice and, therefore, it is suspected to be involved in the pathophysiology of malignant diseases [29]. Anti-A2HSG antibodies have been found in the serum of breast cancer patients [30]. In spite of many diseases and processes of which A2HSG is supposed to be involved in, the exact mechanisms of A2HSG function in (patho)physiology are not known. The connection of A2HSG with cytokines, inflammation, and the bone marrow (and its development) could be of interest in relation to MDS. However, it is difficult to speculate on the possible role of A2HSG in MDS, especially when taking into account that plasma levels may be either decreased or increased due to different conditions [31]. In this study, A2HSG has been identified in four spots, with unique identifications in two of them (spots 3 and 29). Normalized volumes of both the spots were decreased (2.3 and 1.4-fold) in the RA-RARS group. This corresponds to the observed decrease of A2HSG plasma level when absolute quantification was performed (fold 1.2), however, this fold value corresponds rather to 1.4-fold value as observed in the spot 29 than to 2.3-fold in the spot 3. The presence of A2HSG in a few spots changing with different fold values indicates PTM(s); the discrepancy between the changes observed in 2-DE data and absolute quantification further supports this assumption. Finally, relative quantification results showed that while one of the two estimated A2HSG peptides was not altered at all (0.95-fold change), the second peptide was increased more than two-fold in the MDS group (2.54-fold). This also suggests the presence of PTM(s); however, the change was not found to be statistically significant. In a study by Petrik *et al.*[32], A2HSG was found to be a serum marker of survival in patients with glioblastoma; moreover, A2HSG serum level change was not related to comorbidity and inflammation (it has been shown that a decrease in A2HSG levels correlated with the degree of tumor malignancy). The authors also point out that A2HSG was not a glioblastoma specific marker, as there was correlation with the malignancy of other tumor types. As this protein (the decrease in plasma/serum protein levels) mirrors the degree of malignancy found in different tumor types and we have observed decreases of spot normalized volumes in MDS patients, the future task should be profiling the change (and possible correlation) of A2HSG plasma levels in patients with different MDS subgroups, according to their survival and the degree of malignancy. This was not possible to perform yet, due to the limited number of samples. It is also interesting that the change in A2HSG in the study by Petrik *et al.*[32] was found in the A2HSG B-chain, while in our study the A-chain and the connecting peptide were identified (both chains are linked by disulfides, whose bonds are disrupted during 2-DE sample analysis; the B-chain Mw is less than 3 kDa, and therefore could not be detected in our study using 2-DE). As Petrik *et al.* further estimated serum levels using the turbidimetric method and polyclonal antibodies, the decrease in A2HSG serum level should correspond to the whole protein molecule. Nevertheless, the authors also speculated on the possible influence of PTMs (in terms of B-chain and the whole A2HSG molecule); and our study suggests that there could be such a factor (PTM) according to the comparison of 2-DE, absolute and relative quantification results. Another factor that could influence the A2HSG plasma/serum level is proteolytic degradation of the protein; the presence of A2HSG fragments in serum (depending on the protein preparation) has been described in existing literature [22]. However, Petrik *et al.*[32] has proven that the A2HSG level in serum is stable for at least two weeks at 4°C, including after several freeze-thaw cycles.

Retinol-binding protein 4 (RBP4) is a specific carrier protein for retinol [33]; it is associated with variables related to insulin resistance [34]; is involved in inflammation and related to liver function; and its serum level is reduced in critically ill patients [35]. Using 2-DE, a change (decrease) in RBP4 was also observed in the group of chemosensitive patients with multiple myeloma, which were treated with bortezomib-based regimens, when compared with patients resistant to chemotherapy [36], and also in the sera of epithelial ovarian cancer patients [37]. It has also been shown that an increased ratio of unbound to bound (binding to retinol) RBP4 levels may induce apoptosis in renal and endothelial cells [38]. It is known that retinoids (retinol, etc.) are involved in cell differentiation and in tumor development, probably due to the disruption of retinoid signaling [39]. Some anticancer agents may induce RBP4 expression *in vivo*[40]. In our previous proteomic study of the RAEB-1 subgroup [6], RBP4 was identified in a spot with a decreased normalized volume in patients with MDS, compared to healthy controls. Relative label-free quantification of RBP4 peptides was performed; the influence of PTM was not proven; and it was speculated that a RBP4 plasma level change was possibly specific for the RAEB-1 subgroup. In this study, RBP4 was uniquely identified in one spot (spot 30) with its normalized volume decreased in the patient group by 1.4-fold. Thus, the results obtained by 2-DE are in accord with the RAEB-1 subgroup. Nevertheless, when results of relative label-free quantification of RBP4 peptides are compared, some differences may be observed. Relative quantification was performed using the same peptides and conditions as in the previously studied RAEB-1 subgroup. Even though there were no differences between the patient and the healthy control groups observed in both subgroups (i.e., no PTM in the monitored peptides was proven), it is remarkable that there was no difference in peptide levels in RA-RARS observed at all (0.92 and 1.12-fold difference between the patient and control groups), while there was a decrease in the same peptide levels in RAEB-1 (2.33 and 3.25-fold). This strongly indicates an influence of PTM(s) of RBP4 in the RA-RARS subgroup with the position(s) in other (not monitored) peptides. It also suggests that the results observed in the RAEB-1 subgroup were not caused by RBP4 plasma level change only, as it was speculated in the RAEB-1 study [6], but it is likely that there was an influence of both protein modification and level changes. In this study, RBP4 expression estimated by western blot analysis showed no obvious difference. Therefore, western blot analysis supports the notion that RBP4 was influenced by PTM(s). The RBP4 results have shown that although there are almost identical observations using 2-DE, it is necessary to use other methods to ‘see beyond’ as the similar results may be caused by different factors. It has also been shown that mass spectrometry-based relative label-free quantification of peptides may provide additional useful information, in spite of monitoring only a limited number of peptides.

## Conclusions

In conclusion, this is the first report on plasma proteome changes, using 2D electrophoresis, in MDS patients with refractory anemia and refractory anemia with ringed sideroblasts. When comparing results to our previous proteomic studies of other MDS subgroups (refractory cytopenia with multilineage dysplasia and refractory anemia with excess blasts subtype 1), there are agreements in some results, especially in changes in the composition or modification of fragments of inter-alpha-trypsin inhibitor heavy chain H4 protein. Further, agreement in 2D electrophoresis results for retinol-binding protein 4 was observed when compared to our previous study of refractory anemia with excess blasts subtype 1. However, mass spectrometry based relative label-free quantification of retinol-binding protein 4 peptides has shown that there are differences in the PTM(s) of this protein between the two MDS subgroups. The relative label-free quantification (together with absolute quantification and 2D electrophoresis data) also suggests a possible role of PTM(s) of alpha-2-HS-glycoprotein, a protein related to bone marrow development and function, in MDS patients with refractory anemia and refractory anemia with ringed sideroblasts.

## Competing interests

The authors declare that they have no competing interests.

## Authors’contributions

PM and ZRR designed and performed research, analyzed data, and wrote the manuscript. JC diagnosed the patients and collected the samples. KP, JS, JC, and JED designed research and wrote the manuscript. All authors have read and approved the final manuscript.

## Supplementary Material

Additional file 1: Table S1 List of spots that differed significantly when RA-RARS patients and healthy controls were compared. Click here for file
