# Supplementary material for: Plasma proteome changes associated with refractory anemia and refractory anemia with ringed sideroblasts in patients with myelodysplastic syndrome
Source: Proteome Sci. 2013 Apr 8;11:14. doi: 10.1186/1477-5956-11-14 (PMC3635902; doi:10.1186/1477-5956-11-14)
Supplement: Additional file 1: Table S1 — List of spots that differed significantly when RA-RARS patients and healthy controls were compared. [file 1477-5956-11-14-S1.pdf]

Table S1. List of spots that differed significantly when RA-RARS patients and healthy controls were compared.

spot	p	fold	protein	peptides	AC	SC (%)
1	3.7E-05	2.5	Apolipoprotein A-I	5	P02647	28
2	3.9E-07	-2.4	Alpha-2-HS-glycoprotein	3	P02765	13
			Alpha-1-antichymotrypsin	3	P01011	11
			Corticosteroid-binding globulin	2	P08185	14
			Coagulation factor X	2	P00742	6
			Kininogen-1	2	P01042	4
3	0.0001	-2.3	Alpha-2-HS-glycoprotein	2	P02765	7
4	0.004	-2.0	Prothrombin	7	P00734	25
			Insulin-like growth factor-binding protein complex acid labile subunit	3	P35858	13
			Inter-alpha-trypsin inhibitor heavy chain H4	3	Q14624	6
			Histidine-rich glycoprotein	2	P04196	8
			Complement C4-A; (B)	2	P0C0L4;P0C0L5	2
			Extracellular matrix protein 1	2	Q16610	7
5	0.007	2.0	Apolipoprotein A-I	3	P02647	13
			C-reactive protein	2	P02741	16
6	0.009	1.7	Apolipoprotein E	2	P02649	8
7	0.003	-1.7	Apolipoprotein A-IV	2	P06727	12
			Serum albumin	4	P02768	14
8	0.046	1.7	unidentified			
9	0.0007	-1.7	Inter-alpha-trypsin inhibitor heavy chain H4	4	Q14624	6
10	0.001	-1.7	Hemopexin	3	P02790	13
			Beta-2-glycoprotein 1	2	P02749	20
			Kallistatin	2	P29622	11
11	0.029	-1.7	Inter-alpha-trypsin inhibitor heavy chain H4	3	Q14624	4
12	0.002	-1.6	Inter-alpha-trypsin inhibitor heavy chain H4	2	Q14624	3
13	0.0003	-1.6	Hemopexin	3	P02790	18
			Prothrombin	5	P00734	21
			Insulin-like growth factor-binding protein complex acid labile subunit	4	P35858	13
14	0.009	-1.6	unidentified			
15	0.009	-1.6	Apolipoprotein A-IV	2	P06727	7
			Serum albumin	4	P02768	13
16	0.0003	-1.6	Alpha-1-antichymotrypsin	6	P01011	23
			Kininogen-1	7	P01042	16
			Corticosteroid-binding globulin	3	P08185	10
17	0.004	1.6	Prothrombin	7	P00734	30
			Alpha-1-antichymotrypsin	2	P01011	12
18	7.6E-05	-1.5	Vitamin D-binding protein	5	P02774	18
			Angiotensinogen	5	P01019	16
			Antithrombin-III	4	P01008	17
			Thyroxine-binding globulin	3	P05543	10
			Alpha-2-antiplasmin	2	P08697	6
19	0.007	1.5	Apolipoprotein A-I	4	P02647	18
20	0.036	-1.5	Inter-alpha-trypsin inhibitor heavy chain H4	4	Q14624	8
21	0.002	-1.5	Alpha-1-antichymotrypsin	3	P01011	11
			Kininogen-1	3	P01042	13
			Vitronectin	2	P04004	5
22	0.008	1.5	Prothrombin	3	P00734	25
23	0.0003	-1.5	Angiotensinogen	5	P01019	16
			Thyroxine-binding globulin	4	P05543	12
			Alpha-1-antichymotrypsin	3	P01011	11
			Kininogen-1	3	P01042	10
			Alpha-2-antiplasmin	2	P08697	6
24	2.9E-05	-1.5	Angiotensinogen	5	P01019	19
			Antithrombin-III	3	P01008	12
			Thyroxine-binding globulin	3	P05543	13
25	0.021	-1.5	Antithrombin-III	4	P01008	12
			Hemopexin	5	P02790	21
			Beta-2-glycoprotein 1	3	P02749	21
			Complement factor I	2	P05156	6
			Alpha-1-antichymotrypsin	2	P01011	8
			Vitamin D-binding protein	2	P02774	11
26	0.009	-1.5	Complement C1s subcomponent	7	P09871	21
27	0.001	-1.4	Clusterin	3	P10909	10
			Inter-alpha-trypsin inhibitor heavy chain H4	2	Q14624	5
28	0.003	-1.4	Inter-alpha-trypsin inhibitor heavy chain H4	3	Q14624	6
29	0.048	-1.4	Alpha-2-HS-glycoprotein	2	P02765	9
30	0.026	-1.4	Retinol-binding protein 4	3	P02753	39
31	0.033	-1.4	unidentified			
32	0.021	-1.4	Complement factor I	2	P05156	4
33	0.023	-1.4	Alpha-1-antichymotrypsin	4	P01011	14
			Corticosteroid-binding globulin	2	P08185	11
			Kininogen-1	2	P01042	4
34	9.8E-05	-1.4	Kininogen-1	8	P01042	16
			Alpha-1-antichymotrypsin	3	P01011	21
			Angiotensinogen	3	P01019	9
			Vitronectin	2	P04004	5
35	0.003	-1.4	Antithrombin-III	4	P01008	27
			Vitamin D-binding protein	4	P02774	20
			Monocyte differentiation antigen CD14	2	P08571	12
36	0.003	-1.4	Serum amyloid P-component	6	P02743	28
37	0.037	-1.3	unidentified			
38	0.002	-1.3	Alpha-2-HS-glycoprotein	2	P02765	10
			Alpha-1-antichymotrypsin	3	P01011	14
39	0.0003	-1.3	Clusterin	3	P10909	11
40	0.034	-1.3	unidentified			
41	0.004	-1.3	Inter-alpha-trypsin inhibitor heavy chain H4	2	Q14624	6
42	0.032	-1.3	Tetranectin	3	P05452	35
43	0.005	-1.3	Complement factor I	3	P05156	8
			Complement C3	5	P01024	5
			Clusterin	3	P10909	10
44	0.043	-1.3	Hemopexin	7	P02790	24
			Beta-2-glycoprotein 1	5	P02749	21
45	0.002	-1.3	unidentified			
46	0.001	-1.3	Alpha-1-antichymotrypsin	10	P01011	32
			Kininogen-1	2	P01042	4
47	0.032	-1.3	Serum albumin	17	P02768	45
			Hemopexin	2	P02790	9
48	0.002	-1.3	Clusterin	5	P10909	19
49	0.008	-1.3	Clusterin	3	P10909	17
50	0.019	1.2	Protein AMBP	3	P02760	20
51	0.024	-1.2	Complement component C9	4	P02748	13
			Alpha-2-antiplasmin	4	P08697	13
			Apolipoprotein A-IV	2	P06727	11
			Alpha-1-antichymotrypsin	2	P01011	11
			Vitamin D-binding protein	3	P02774	19
			Antithrombin-III	3	P01008	8
			Kininogen-1	2	P01042	10
52	0.005	-1.2	Alpha-1B-glycoprotein	5	P04217	30
			Prothrombin	7	P00734	23
			Afamin	5	P43652	15
			Apolipoprotein A-IV	3	P06727	12
			Inter-alpha-trypsin inhibitor heavy chain H4	4	Q14624	4
			Complement C5	3	P01031	5
			Insulin-like growth factor-binding protein complex acid labile subunit	4	P35858	13
			Vitamin D-binding protein	3	P02774	11
			Vitronectin	2	P04004	9
53	0.001	-1.2	Antithrombin-III	12	P01008	30
			Vitamin D-binding protein	15	P02774	49
			Angiotensinogen	6	P01019	17
			Apolipoprotein A-IV	3	P06727	12
			Monocyte differentiation antigen CD14	2	P08571	9
			Alpha-2-antiplasmin	3	P08697	12
54	0.027	1.2	Protein AMBP	3	P02760	14
55	0.031	-1.2	Prothrombin	11	P00734	34
			Alpha-1B-glycoprotein	5	P04217	25
			Inter-alpha-trypsin inhibitor heavy chain H4	7	Q14624	11
			Afamin	3	P43652	12
			Insulin-like growth factor-binding protein complex acid labile subunit	2	P35858	8

p - ANOVA p-value; **fold** - fold difference (multiplication) when RA-RARS patients and healthy controls compared (+fold when the normalized volumes increased in RA-RARS); **protein** - protein identification; **peptides** - number of unique peptides fulfilling a minimal Mascot score for identity; **AC** - accession number (SWISS-PROT); **SC** - protein sequence coverage
